# Turtle interacts with borderless in regulating glial extension and axon ensheathment

**DOI:** 10.1186/s13041-017-0299-6

**Published:** 2017-05-23

**Authors:** Yixu Chen, Scott Cameron, Wen-Tzu Chang, Yong Rao

**Affiliations:** 1 McGill Centre for Research in Neuroscience, Department of Neurology and Neurosurgery, McGill University Health Centre, 1650 Cedar Avenue, Montreal, QC H3G 1A4 Canada; 2 Integrated Program in Neuroscience, McGill University Health Centre, 1650 Cedar Avenue, Montreal, QC H3G 1A4 Canada; 3 Department of Medicine, McGill University Health Centre, 1650 Cedar Avenue, Montreal, QC H3G 1A4 Canada; 4 Centre for Research in Neuroscience, McGill University Health Centre, Room L7-136, 1650 Cedar Avenue, Montreal, QC H3G 1A4 Canada

## Abstract

Proper recognition between axons and glial processes is required for the establishment of axon ensheathment in the developing nervous system. Recent studies have begun to reveal molecular events underlying developmental control of axon-glia recognition. In our previous work, we showed that the transmembrane protein Borderless (Bdl) is specifically expressed in wrapping glia (WG), and is required for the extension of glial processes and the ensheathment of photoreceptor axons in the developing *Drosophila* visual system. The exact mechanism by which Bdl mediates axon-glia recognition, however, remains unknown. Here, we present evidence showing that Bdl interacts with the Ig transmembrane protein Turtle (Tutl). Tutl is specifically expressed in photoreceptor axons. Loss of *tutl* in photoreceptors, like loss of *bdl* in WG, disrupts glial extension and axon ensheatment. Epistasis analysis shows that Tutl interacts genetically with Bdl. Tutl interacts with Bdl in trans in cultured cells. We propose that Tutl interacts with Bdl in mediating axon-glia recognition for WG extension and axon ensheathment.

## Introduction

The ensheathment of axons by glial processes is required for insulating axons for propagating action potentials [1]. The establishment of axon ensheathment during development requires proper recognition between nascent axons and glial processes. While recent studies have made progress in identifying factors important for axon ensheathment, the molecular mechanisms underlying developmental control of axon-glia recognition are still poorly defined.

Recent data from genetic dissection of the development of the *Drosophila* adult visual system reveals several important genes in the control of glial development and axon ensheathment. For instance, the FGF signaling pathway has been implicated in regulating glial proliferation, migration and differentiation [2]. At the third-instar larval stage, activation of the receptor tyrosine kinase Heartless (Htl) by the FGF8-like ligand Pyramus promotes the proliferation of perineurial glia (PG) and subsequent migration of PG from the optic stalk into the eye disc. PG migration also requires Gilgamesh, Hedgehog and Integrin [3, 4]. Upon reaching the eye disc, Htl is then activated by the neuron-derived FGF8-like ligand Thisbe, which promotes the differentiation of PG into wrapping glia (WG) and the initiation of photoreceptor (R cell) axon ensheathment [2].

The mechanisms underlying axon-glia recognition for R-cell axon ensheathment remains unclear. In our previous study [5], we identified the immunoglobulin-like (Ig) transmembrane protein Borderless (Bdl) as a key player in regulating WG extension and R-cell axon ensheathment. The expression of Bdl undergoes dynamic changes during visual circuit development. At later stage (i.e. pupal stage), Bdl is expressed in R-cell axons, and down-regulation of Bdl is required for R7 axonal tiling [6]. Whereas at earlier stage (i.e. third-instar larval stage), Bdl is exclusively expressed in WG and is specifically required for WG extension and R-cell axon ensheathment [5]. Those data leads to the speculation that Bdl mediates axon-glia recognition at the third-instar larval stage by interacting with an unknown receptor on differentiating R-cell axons.

To determine the exact mechanism by which Bdl mediates axon-glia recognition, we set out to identify R-cell axon-surface factors that interact with Bdl on WG in the developing fly visual system. We found that mutants defective in the *turtle* (*tutl*) gene encoding for a transmembrane protein, a member of the conserved IgSF9/Dasm1/Tutl subfamily of the Ig superfamily [7–10], displayed a *bdl*-like WG extension and axon ensheathment phenotype. Further molecular and genetic analysis support that WG-specific Bdl interacts with Tutl on R-cell axons in mediating axon-glia recognition for WG extension and axon ensheathment.

## Results

### Tutl is expressed in R-cell axons in the developing visual system at third-instar larval stage

In our previous study [7], we showed that Tutl is present on R-cell axons and is required for specifying R7 axonal tiling at later stages (i.e. pupal stage). To determine if Tutl also plays a role in regulating visual circuit development at earlier stage (i.e. third-instar larval stage), we examined the expression pattern of Tutl in third-instar larval eye-brain complexes. At the third-instar stage, differentiating WG processes in the eye disc contact nascent R-cell axons, and subsequently migrate along R-cell axons from the eye disc through the optic stalk into the optic lobe.

**Fig. 1:**
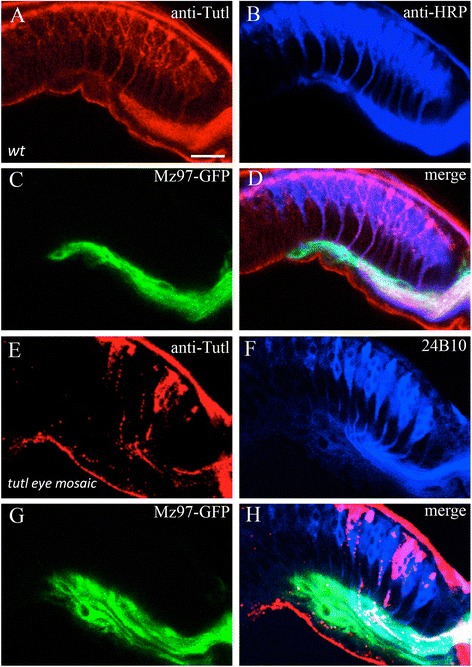
Tutl is expressed in R cells in the developing visual system at third-instar larval stage. Longitudinal optic sections of wild-type (**a**-**d**) and *tutl*-eye mosaic (**e**-**h**) third-instar eye discs were triple-stained. Tutl immunoreactivity was detected with a rabbit anti-Tutl antibody (*red*). WG processes were visualized with UAS-mCD8-GFP driven by the WG-specific Mz97-GAL4 (i.e. Mz97-GFP) (*green*). R-cell bodies and their axons were labeled with anti-HRP (**b**) or MAb24B10 (**f**) (*blue*). Apical is up, and posterior is to the right. **a** In wild type, Tutl immunoreactivity was detected in R-cell bodies and axons in the eye epithelium as well as subretinal regions where R-cell axons migrate posteriorly towards the optic stalk. **b** All R-cell bodies and axons in **a** were visualized with anti-HRP staining. **c** WG processes in **a** were labeled with Mz97-GFP. WG processes at the subretinal region follow R-cell axons and migrate posteriorly. **d** Images in **a**-**c** were merged. (**e**-**h**) Large clones of homozygous *tutl* ^23^ mutant eye tissues were generated with eye-specific mitotic recombination. Thus, the *tutl* gene was specifically removed in mutant eye clones, but not removed in WG. **e** Anti-Tutl staining. Tutl immunoreactivity was absent in mutant R-cell bodies and axons. At subretinal regions where *tutl* mutant R-cell axons associate with WG processes, Tutl immunoreactivity was also missing. **f** All R-cell bodies and axons in **e** were labeled with MAb24B10 staining. **g** WG processes in **e** were labeled with Mz97-GFP. **h** Images in **e**-**g** were merged. Scale bar: 10 μm

In wild type, anti-Tutl staining was detected in R-cell bodies and axons (Fig. 1a, d). To confirm the specificity of anti-Tutl staining, we performed anti-Tutl staining of *tutl* eye-mosaic mutants in which the majority of eye tissues were homozygous for *tutl* mutations. Since *tutl* mutant clones were generated by eye-specific mitotic recombination, the *tutl* gene was selectively removed in R cells, but not removed in glia. We found that anti-Tutl staining was absent in *tutl* mutant R cells in the eye disc, and was also missing in subretial space where *tutl* mutant axons projected into, while R-cell differentiation remains normal in *tutl* eye-mosaic tissues (Fig. 1e and h). This result indicates that unlike Bdl that is exclusively expressed in WG [5], Tutl is specifically expressed in R-cell bodies and axons.

### *tutl* is required for the extension of WG processes along R-cell axons in the optic lobe

In our previous study [5], we showed that loss of *bdl* caused defects in the extension of WG processes along R-cell axons within the optic lobe. We speculated that Bdl on WG recognizes an unknown receptor on R-cell axons for promoting WG extension and axon ensheathment. Since Tutl is present on R-cell axons at the third-instar larval stage when WG initiates its contact with R-cell axons (Fig. 1), we tested Tutl as one of the candidate receptors on R-cell axons that may be recognized by Bdl on WG.

**Fig. 2:**
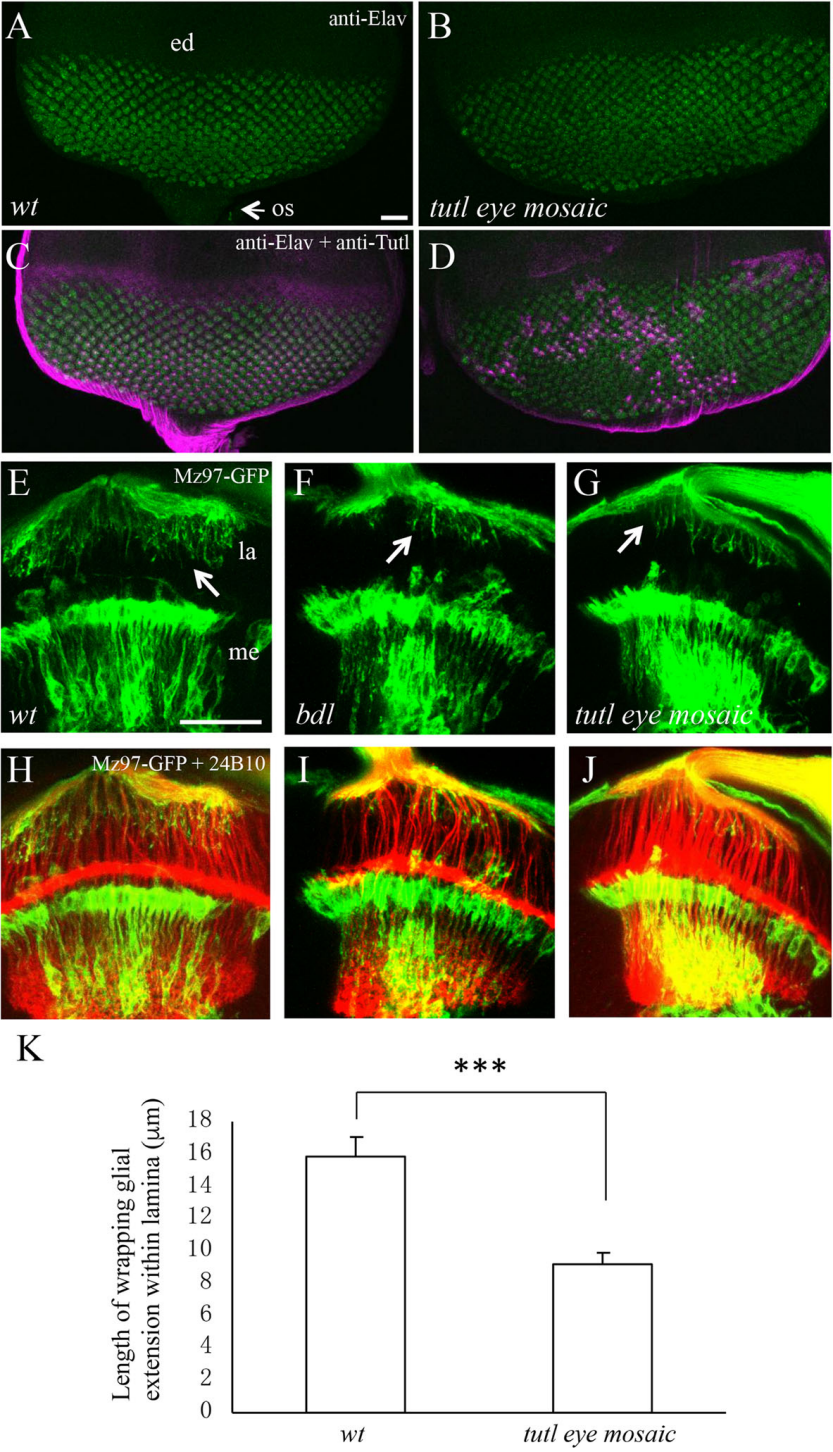
Loss of *tutl* in R cells disrupts the extension of WG processes in the optic lobe. **a**-**d** Third-instar eye discs were double-stained with anti-Elav (*green*) and anti-Tutl (magenta). Anti-Elav antibody recognizes the neuronal-specific nuclear protein Elav that is expressed in all differentiating R cells. Anterior is up. **a** and **c** In wild type, differentiating R-cell nuclei were observed in the posterior region of the eye disc. **b** and **d** In *tutl* ^23^ eye-specific mosaic individuals, R-cell differentiation occurred normally. Homozygous *tutl* mutant tissues were identified by lack of anti-Tutl staining. **e**-**j** Third-instar eye-brain complexes were double-stained with mCD8-GFP (*green*) and MAb 24B10 (*red*). **e** and **h** In wild type, WG in the subretinal region project processes (arrow) that follow R-cell axons from the eye disc through the optic stalk (os) into the lamina (la). **f** and **i** In homozygous *bdl* ^EX2^ mutants, much less and shorter WG glial processes followed R-cell axons into the lamina. **g** and **j** In *tutl* ^23^ eye-specific mosaic individuals in which *tutl* was specifically removed in R cells, but not in WG, a *bdl*-like WG extension phenotype was observed. Note that R-cell axons projected normally into the optic lobe in *tutl* mosaic individuals (**j**). **k** The length of individual WG extension within lamina was measured. Loss of *tutl* in R cells significantly affected the extension of WG processes (*p* < 0.001). Two-tailed t-tests were used. Error bars indicate SEM. Abbreviations: ed, eye disc; os, optic stalk; la, lamina; me, medulla. Number of individuals examined: wild type, 9; *tutl* eye-mosaic, 10. Scale bar: **a**-**d**, 10 μm; **e**-**i**, 20 μm

To determine the potential role of Tutl in WG extension, we examined the effects of removing *tutl* on R-cell projections and WG extension. To specifically remove *tutl* in R cells, we performed eye-specific genetic mosaic analysis. Large clones (>90% of eye tissues) of homozygous *tutl* tissues were generated in the developing eye. This allows the removal of the *tutl* gene in R cells, but not in WG. The patterns of R-cell axonal projection and WG extension in *tutl* eye-mosaic mutants were then analyzed. In wild type, developing R cells in the eye disc project their axons basally into subretinal space, and then migrate posteriorly into the optic stalk (Figs. 1d and 2a). After exiting the optic stalk, R-cell axons enter the lamina, the superficial layer of the developing optic lobe (Fig. 2h). WG processes contact the nascent R-cell axons at the subretinal space, and subsequently extend along the surface of R-cell axons from the eye disc through the optic stalk into the optic lobe (Figs. 1d and 2h).

Compared to that in wild type (Fig. 2a and c), R-cell differentiation occurred normally in the posterior region of the eye disc in *tutl* eye-mosaic mutants (Fig. 2b and d). Like that in wild type (Fig. 2h) and *bdl* mutants (Fig. 2i), R-cell axons in *tutl* eye-mosaic mutants projected normally into the optic lobe (Fig. 2j). *tutl* R1-R6 growth cones established a smooth and continuous layer at the lamina termination site, their intermediate target region, while *tutl* R7 and R8 axons projected normally into the deeper medulla layer (Fig. 2j). However, we found that like that in *bdl* mutants (Fig. 2f, i), much less and shorter WG processes were observed in the lamina in *tutl* eye-mosaic mutants (Fig. 2g, j, k). These results indicate that *tutl*, like *bdl*, is required for the extension of WG processes in the lamina.

### Eye-specific knockdown of *tutl* also disrupts glial extension

**Fig. 3:**
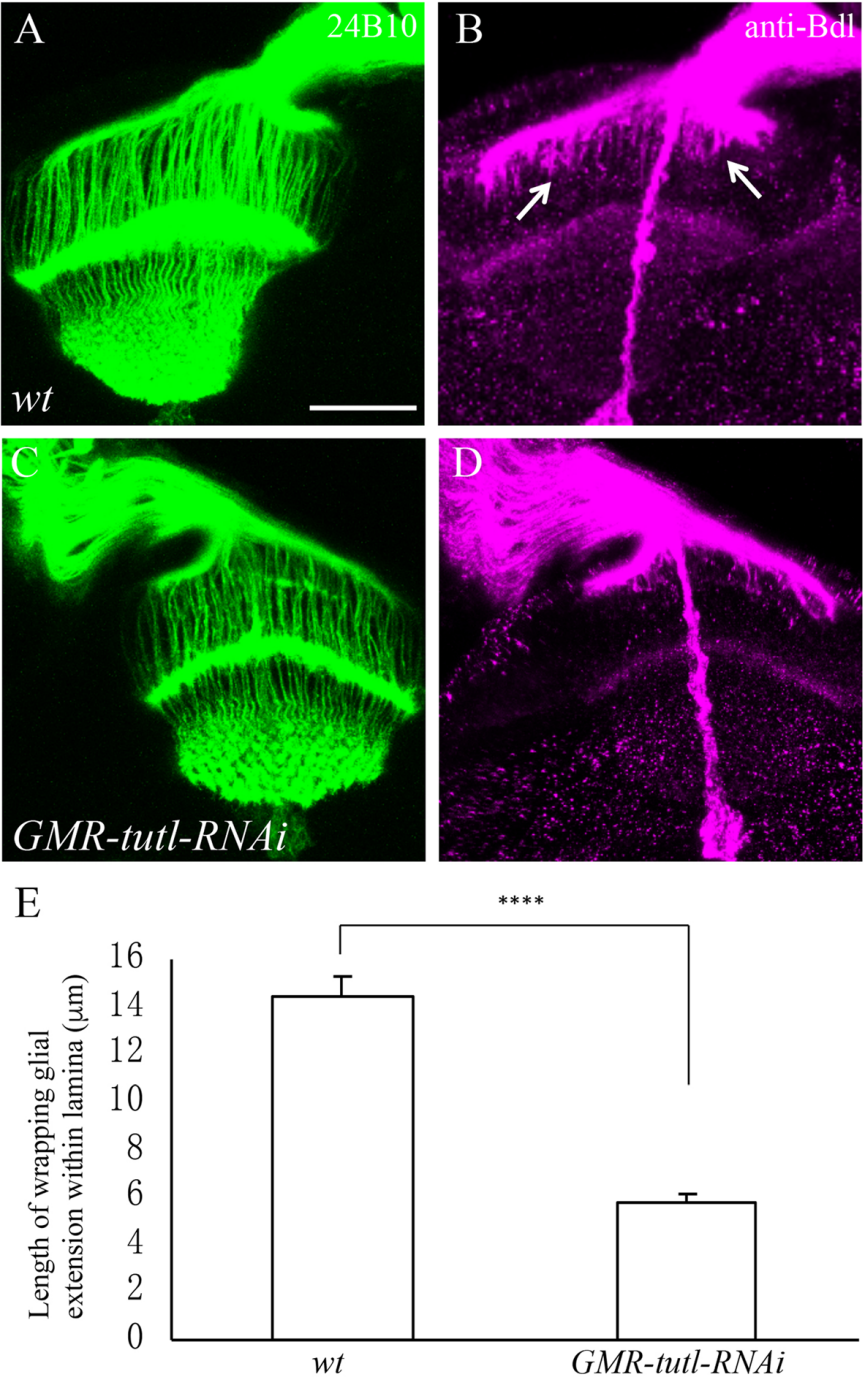
Knockdown of *tutl* in R cells also disrupts glial extension. **a**-**d** WG processes and R-cell axons in third-instar eye-brain complexes were visualized with anti-Bdl (magenta) and MAb 24B10 (*green*), respectively. **a** and **b** In wild type, WG processes follow R-cell axons into the lamina (*arrows*). **c** and **d** When *tutl* was knocked down in R cells, but not in WG, WG extension in the lamina was disrupted. GMR-*tutl-RNAi* denotes that the UAS-*tutl-RNAi* transgene was under control of the eye-specific driver GMR-GAL4. **e** The length of individual WG extension within lamina was measured. Knockdown of *tutl* in R cells significantly disrupted WG extension (*p* = 2.17E-07). Two-tailed t-tests were used. Error bars indicate SEM. Number of individuals examined: wild type, 9; GMR-*tutl-RNAi*, 12. Scale bar: 20 μm

We also examined the effects of knocking down *tutl* on the extension of WG by utilizing an UAS*-tutl-RNAi* transgene, which was shown to effectively reduce the level of *tutl* in R cells in the our previous study [7]. The expression of UAS*-tutl-RNAi* was controlled by the eye-specific driver GMR*-*GAL4, which allows the expression of UAS*-tutl-RNAi* in R cells, but not in glia. Since Bdl is exclusively expressed in WG at this stage, we performed anti-Bdl staining to examine WG extension. Although knockdown of *tutl* in R cells did not affect the expression level of Bdl in WG (data not shown), the extension of WG in the lamina was severely disrupted (Fig. 3c, d, e). This result, together with that from eye-specific mosaic analysis (Fig. 2), indicates a role for *tutl* in R-cell axons for promoting WG extension in the developing optic lobe.

### *tutl* interacts with *bdl* genetically in the control of WG extension

That loss of *tutl* in R-cell axons phenocopies that caused by loss of *bdl* in WG, suggests that Tutl interacts with Bdl in mediating axon-glia recognition for promoting WG extension. To further assess this, we performed epistasis analysis in two ways.

**Fig. 4:**
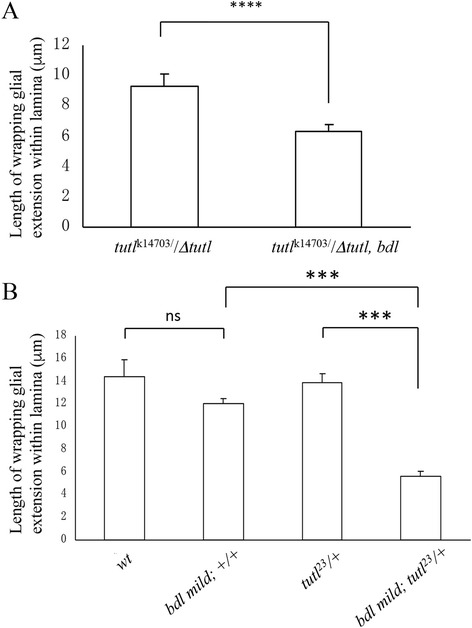
*tutl* interacts genetically with *bdl* in the control of WG extension. **a** Reducing the dosage of *bdl* enhanced the WG extension phenotype in *tutl* ^k14703^ hemizygotes. Flies in which the *tutl* ^k14703^ allele was placed over a *tutl* deletion allele (i.e. *tutl* ^23^), showed a mild WG extension phenotype. This *tutl* ^k14703^ hemizygote phenotype was significantly enhanced when the dosage of *bdl* was reduced by 50%. “****”, *p* < 0.00001. Two-tailed t-tests were used. Number of individuals examined: *tutl* ^k14703^/Δ*tutl*, 9; *tutl* ^k14703^/Δ*tutl, bdl*, 12. **b** Reducing the dosage of *tutl* enhanced the WG phenotype induced by mild overexpression of *bdl* in WG. Mild *bdl* overexpression in WG (i.e. *bdl* mild) did not affect WG extension. However, *bdl* mild-overexpression flies in which the dosage of *tutl* was reduced by 50%, displayed a strong WG extension phenotype. ns, not significant; “***”, *p* < 0.0001. One-way ANOVA analysis was used. Error bars indicate SEM. Number of individuals examined: wild type, 9; *bdl* mild; +/+, 13; *tutl* ^23^/+, 6; *bdl* mild; *tutl* ^23^/+, 15

Firstly, we tested the effects of reducing the dosage of *bdl* on the WG extension phenotype caused by partial loss of *tutl*. Weak *tutl* mutants (i.e. *tutl* ^k14703^/Δ*tutl*) displayed a mild WG extension phenotype (Fig. 4a). When the dosage of *bdl* was reduced by 50%, we found that the WG extension phenotype was significantly enhanced (Fig. 4a).

And secondly, we examined the effects of reducing the dosage of *tutl* on WG extension in flies in which *bdl* was mildly overexpressed in WG. In our previous study [5], we showed that strong overexpression of Bdl in WG disrupted WG extension by increasing WG-WG homotypic adhesion, whereas mild overexpression of Bdl in WG did not affect WG extension. We reasoned that if Tutl on R-cell axons indeed interacts with Bdl on WG, then decreasing the level of Tutl on R-cell axons may free some Tutl-interacting Bdl for Bdl-Bdl homophilic binding on opposing WG surface, thus enhancing WG-WG adhesion. Interestingly, we found that although mild Bdl overexpression did not cause a WG extension phenotype (Fig. 4b), reducing the dosage of *tutl* by 50% in flies with mild *bdl* overexpression severely disrupted WG extension (Fig. 4b).

Taken together, above results support that Tutl and Bdl interact in vivo in regulating WG extension.

### Blockade of FGF8 signaling in WG severely disrupts glial extension

**Fig. 5:**
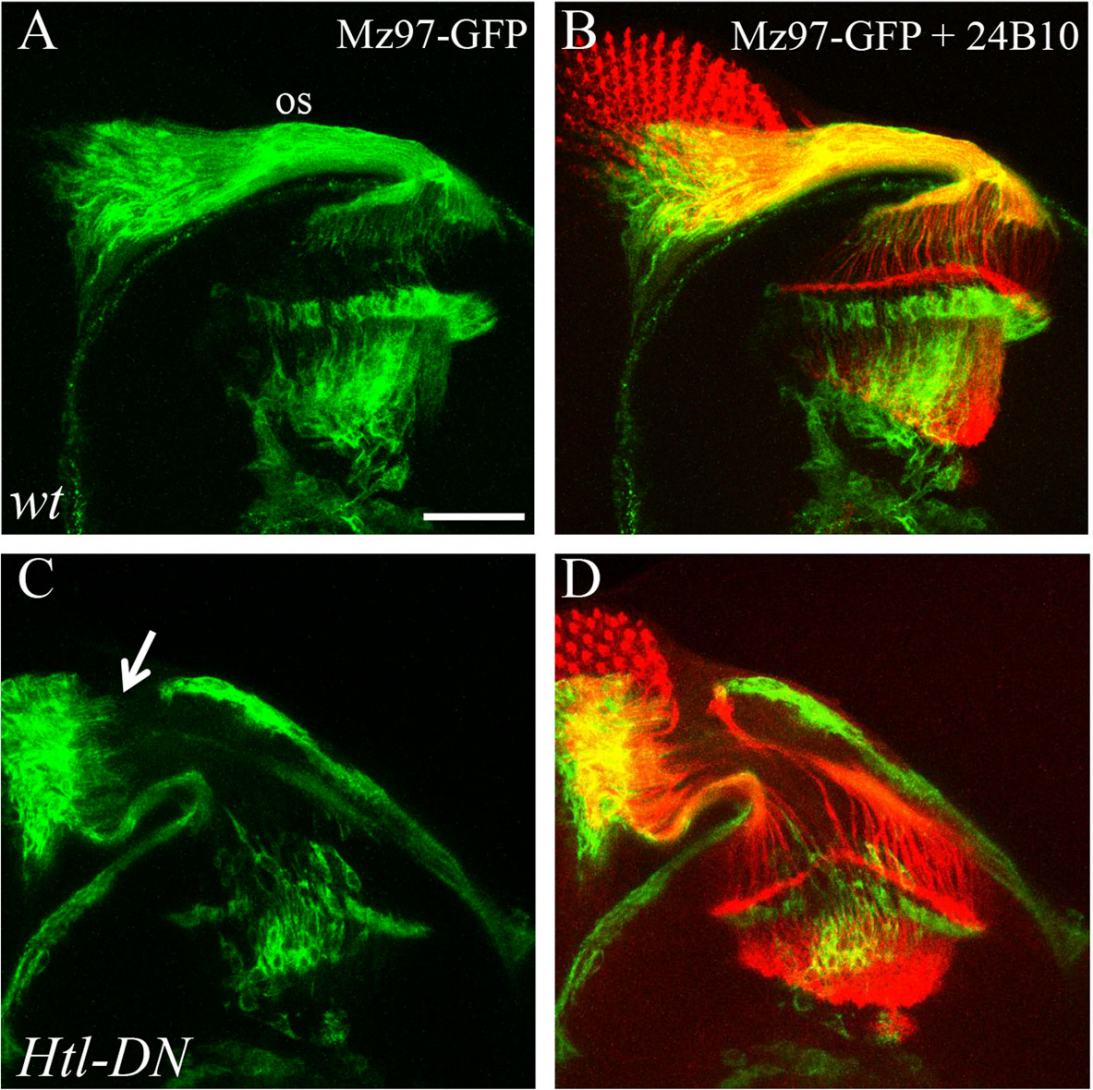
Blockade of FGF8 signaling in WG prevents WG processes from entering the optic stalk. Third-instar eye-brain complexes were double-stained with mCD8-GFP (*green*) and MAb 24B10 (*red*). **a** and **b** Wild type. **c** and **d** In flies with the expression of a dominant-negative form of *htl* under control of the WG-specific driver Mz97-GAL4 (*n* = 35), WG extension was severely disrupted. In flies showing the most severe phenotype (~14%), the majority of WG processes failed to enter the optic stalk (*arrow*), a phenotype that was never observed in *bdl* or *tutl* null mutants. “os” denotes optic stalk. Scale bar: 20 μm

Previous studies by us and others show that the FGF8 receptor Heartless (Htl) is required for WG extension and axonal ensheathment [2, 5]. However, we did not detect any genetic interaction between *bdl* and *htl* [5]. To further evaluate the role of Htl in WG extension, we examined the effects of blocking the function of Htl specifically in WG. A dominant-negative form of Htl that has been shown previously to block the Htl activity effectively [2, 11], was expressed in WG under control of the WG-specific driver Mz97-GAL4. We found that interfering with the Htl function with this dominant-negative form caused a much more severe phenotype (Fig. 5c and d). The majority of WG processes failed to enter the optic stalk. This phenotype was never observed in *tutl* or *bdl* null mutants. Similarly, no such phenotype was observed even when the level of *tutl* was reduced in *bdl* null mutants (*n* = 51). This result suggests a role for Htl in WG extension that is different from that of Tutl and Bdl.

### Loss of *tutl* disrupts the ensheathment of R-cell axons

In our previous study [5], we showed that loss of *bdl* disrupted WG extension and the ensheathment of R-cell axons. To determine if *tutl* mutations cause a similar phenotype in R-cell axonal wrapping, we performed ultrastructural analysis to examine R-cell axon ensheathment in the optic stalk at third-instar larval stage.

**Fig. 6:**
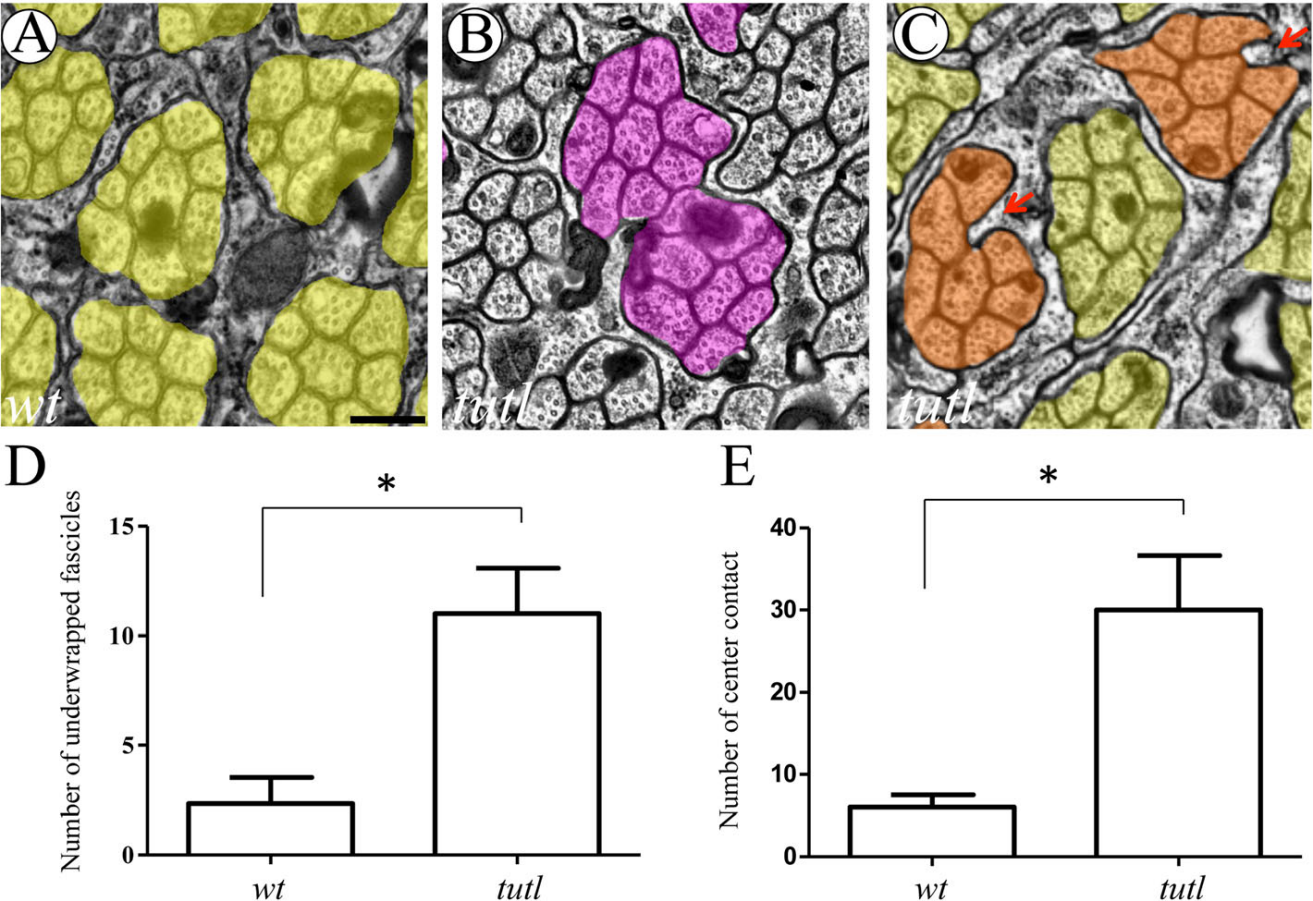
Loss of *tutl* in R cells disrupts axon ensheathment. **a**-**d** Third-instar larval optic stalks were analyzed by electron microscopy. False coloring indicates R-cell axonal profile. **a** In wild type, eight R-cell axons from the same ommatidia form a single bundle, which is wrapped by WG processes within the optic stalk. Note that the central R8 axon is surrounded by other R-cell axons, and does not contact WG processes. **b** and **c** In *tutl* ^01085^ eye-specific mosaic individuals, in which *tutl* was mutated in R cells but not in WG, two types of defects in axonal ensheatment were observed. First, some R-cell axonal fascicles were not separated by WG processes (**b**). And second, some WG processes extended aberrantly within the fascicle to contact the central R8 axon (arrows) (**c**). **d** The phenotype in **b** was quantified. Compared to that of wild type, loss of *tutl* in R cells significantly increased the number of fascicles that were not separated by glial processes (*, *p* < 0.05). **e** The phenotype in **c** was quantified. Loss of *tutl* in R cells led to ectopic extension of glia processes that contact the central R8 axon (*, *p* < 0.05). Two-tailed t-tests were used. For each genotype, three optic stalks were analyzed by electron microscopy. Error bars indicate SEM. Scale bar: 200 nm

In wild type, R-cell axons project from the eye disc into the optic stalk where R-cell axons are wrapped by WG processes (Fig. 6a). R-cell axons from the same ommatidia form a single bundle in which the central R8 axon is surrounded by R1-R7 axons. Each mature R-cell axonal bundle is ensheathed by WG processes that follow R-cell axons from the eye disc into the optic stalk. To assess the potential role of *tutl* in axon ensheathment, we performed eye-specific genetic mosaic analysis. Large clones (>90% of eye tissues) of homozygous *tutl* eye tissues were generated, which removed *tutl* in R cells, but not in glia. Interestingly, we found that like loss of *bdl* in WG [5], loss of *tutl* in R-cell axons caused defective entheathment of R-cell axons in the optic lobe. Compared to that in wild type, the number of bundles completely wrapped by glial processes decreased significantly in *tutl* eye-mosaic animals (Fig. 6b, d), a phenotype identical to loss of *bdl* [5]. Additionally, like loss of *bdl* in WG [5], loss of *tutl* in R-cell axons also caused some glial processes to extend into the interior region of the fascicle to contact the central R8 axon (Fig. 6c, e). We conclude that Tutl, like Bdl, is required for R-cell axonal ensheathment.

### Bdl displays trans interactions with Tutl in cultured cells

That removal of *tutl* in R-cell axons or loss of *bdl* in WG causes a similar WG extension and axon ensheathment phenotype, together with the observed genetic interactions between *tutl* and *bdl*, raise the interesting possibility that Bdl on WG interacts with Tutl on R-cell axons in trans in mediating axon-glia recognition.

**Fig. 7:**
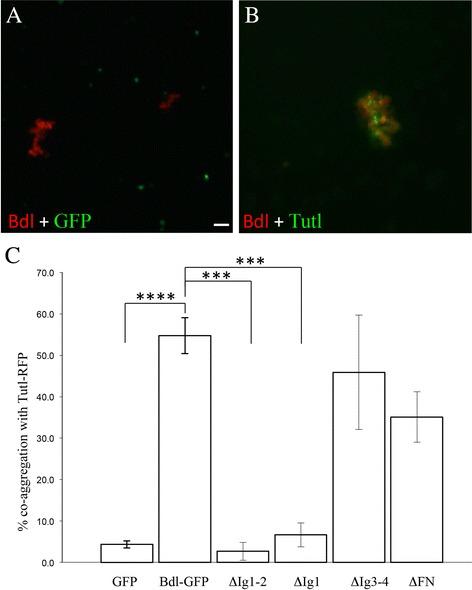
Bdl interacts with Tutl in trans in cultured cells. **a** Bdl-RFP-transfected S2 cells (*red*) formed large cell aggregates with each other, but not with cells transfected with a GFP construct (*green*). **b** Bdl-RFP-transfected S2 cells (red) formed large co-aggregates with cells transfected with a Tutl-GFP construct (*green*). **c** Among total cell aggregates (>20 cells), the percentage of cell co-aggregates were quantified. A cell co-aggregate is defined as a cell aggregate (>20 cells) in which the percentage of each cell population (i.e. GFP- or RFP-positive cells) should be at least 15%. Three independent experiments were performed. Error bars indicate SEM. “****”, *p* < 0.0001; “***”, *p* < 0.001. Two-tailed t-tests were used. Scale bar: 20 μm

To test if Bdl is capable of interacting with Tutl in trans on cell surface, we performed cell-cell co-aggregation studies. Bdl and Tutl were expressed in *Drosophila* Schneider-2 cells (S2 cell). In our previous study [6], we showed that Bdl possesses homophilic binding activity and could induce the formation of large cell aggregates (>20 cells) (Fig. 7a and c). When two populations of cells were mixed, Bdl-RFP-transfected cells selectively formed large aggregates with each other, but not with GFP-transfected cells. Interestingly, we found that Tutl-GFP-transfected cells also formed co-aggregates with Bdl-RFP-transfected cells (Fig. 7b and c). We then tested a set of Bdl deletion constructs in which certain Ig-like domains or fibronectin-type-III repeats (FN) are deleted. Deletion of both Ig-like domain 1 and 2, or deletion of Ig-like domain 1, prevented the co-aggregation of Bdl-transfected cells with Tutl-transfected cells (Fig. 7c). By contrast, deletion of Ig-like domain 3 and 4, or deletion of two FN repeats, did not affect cell-cell co-aggregation. These results indicate that Bdl is capable of interacting with Tutl in trans, and this trans interaction requires the Ig-like domain 1 of Bdl.

## Discussion

Our previous study suggests that Bdl functions as a WG-specific cell surface receptor in mediating WG extension and axon ensheathment by interacting with an unknown ligand on R-cell axons [5]. In this study, we present several lines of evidence that supports that Tutl functions as a Bdl-interacting partner on R-cell axons. Tutl is expressed in R cells at third-instar larval stage. Loss of *tutl* in R-cell axons, like loss of *bdl* in WG, disrupts WG extension and axon ensheathment. Tutl displays trans interactions with Bdl in cultured cells, and interacts genetically with Bdl in the control of WG extension and axon ensheathment in the developing visual system. We propose that Bdl on WG interacts with Tutl on R-cell axons in mediating axon-glia recognition in the developing visual system.

Our results identify a novel and important role for Tutl in mediating glial extension and axon ensheathment at third-instar larval stage. Loss of *tutl* disrupts WG extension and R-cell axon ensheathment, but does not affect R-cell differentiation and axonal projections. In our previous study [7], we showed that Tutl plays an important role in specifying R7 axonal tiling at later stages (i.e. pupal stage). In addition to its role in the developing visual system, Tutl has also been shown to act in the embryonic nervous system to regulate midline crossing, dendritic morphogenesis and larval locomotion [12–14].

In addition to its interaction with Tutl, Bdl also possesses homophilic binding activity [6]. In our previous study [5], we showed that strong overexpression of Bdl in WG also disrupts glial extension by increasing glia-glia adhesion. Interestingly, reducing the level of *tutl* enhanced the phenotype in flies with mild Bdl overexpression (Fig. 5). One possible explanation is that a reduction in the level of Tutl on R-cell axons frees some Bdl on WG from the Bdl-Tutl trans interaction, and thus increases the Bdl-Bdl-mediated glia-glia adhesion leading to an enhancement of the WG extension phenotype. It appears likely that proper WG extension requires a fine balance between Bdl-Tutl-mediated axon-glia recognition and Bdl-Bdl-mediated glia-glia adhesion.

The Bdl-Tutl-dependent recognition system is unlikely to be the only axon-glia recognition system that mediates WG extension and axon ensheathment in the developing visual system. Loss of *tutl*, or loss of *bdl*, does not completely block WG extension. Moreover, in *tutl* and *bdl* mutants, many R-cell axonal bundles are still wrapped correctly by glial processes within the optic stalk. These observations support the existence of other axon-glia recognition systems that function together with Bdl and Tutl in mediating WG extension and axon ensheathment in the developing visual system.

Recent studies show that activation of the FGF8 signaling pathway in WG is also required for WG extension and R-cell axonal ensheathment in the fly visual system [2, 5]. However, several lines of evidence suggest that the FGF8 signaling pathway functions differently from Tutl and Bdl in promoting WG extension and axon ensheathment. First, blockade of FGF8 signaling in WG causes a much more severe WG extension phenotype than that in *tutl* or *bdl* null mutants (Fig. 5). Second, loss of *tutl*, like loss of *bdl*, causes some glial processes to extend into the interior region of R-cell axonal bundles to contact the central R8 axon (Fig. 6) [5], a phenotype that was not observed in mutants defective in FGF8 signaling [2]. And third, no genetic interaction between Bdl and the FGF8 receptor Htl was detected [5]. Those results raise several possibilities. For instance, the FGF signaling pathway functions in parallel to the Tutl-Bdl-mediated recognition system. Alternatively or additionally, FGF8 signaling may modulate the activity of Bdl as well as other Tutl-Bdl-independent recognition systems. Future studies will be needed to distinguish among these possibilities.

Recent studies show that Dasm1/IgSF9A and IgSF9B, the homologs of Tutl in mammals, function in the mammalian developing nervous system. IgSF9B promotes inhibitory synapse development in cultured cells [15], whereas knockout IgSF9/Dasm1 impairs inhibitory synapse development [16]. It will be of interest to determine if Dasm1/IgSF9A and IgSF9B also play a similar role in mediating axon-glia recognition in mammals.

## Methods

### Genetics

*tutl* ^01085^ (BDSC#10979), *tutl* ^K14703^ (BDSC#10451), UAS-*htl. DN* (BDSC#5366) were obtained from the Bloomington Drosophila Stock Center. UAS-*tutl-RNAi* transgene and the *tutl* ^23^ allele (i.e. Δ*tutl*) in which most of the *tutl* genomic sequence was deleted, were generated in our previous study [7, 14]. *bdl* ^EX2^ and *tutl*/*bdl* double deletion (i.e. Δ*tutl, bdl*) alleles were generated in our previous study [6]. Large clones (>90% of retina) of *tutl* ^01085^ or *tutl* ^23^ mutant tissues were generated in an otherwise heterozygous or wild-type eye by eye-specific mitotic recombination using the *ey*Flp*/FRT* system [17]. For knocking down *tutl* in R cells, UAS-*tutl*-RNAi flies were crossed with flies carrying the eye-specific driver GMR-GAL4. To overexpress Bdl in WG, flies carrying the WG-specific driver Mz97-GAL4 were crossed with flies carrying the mild UAS-*bdl* transgene generated in our previous study [5]. For the blockade of the Htl activity in WG, yw; UAS-*htl. DN* flies were crossed with flies carrying the WG-specific driver Mz97-GAL4.

### Molecular biology

UAS-*tutl-GFP* and UAS-*bdl-RFP* were generated in our previous studies [6, 7]. Bdl domain deletion constructs were generated in our previous study [6], including ΔIg1 (Asn27 to Ser128), ΔIg2 (Ser128 to Ile233), ΔIg1-2 (Asn42 to Ile233), ΔIg3-4 (Tyr259 to Ala415), and ΔFN1-2 (Ala434 to Ser636).

### Histology

Whole mount eye–brain complexes from third-instar larvae were dissected and stained as described previously [18, 19]. Antibodies were used at following dilutions: mouse MAb24B10 (1:100; Developmental Studies Hybridoma Bank or DSHB Cat#24B10), Anti-Elav antibody (1:100, DSHB Cat#Elav-9F8A9), rabbit polyclonal anti-GFP (1:750; Molecular Probes, Cat#A-11122), rabbit polyclonal anti-Tutl (1:60000) [6, 7], rabbit polyclonal anti-Bdl (1:1000) [6, 7], alexa 647-conjugated goat anti-HRP (1:500; Jackson ImmunoResearch Cat#123-605-021). Secondary antibodies including CF543 Donkey anti-Mouse IgG (Biotium, Cat#20305) and Alexa Fluor 647 Goat anti-Rabbit IgG (Invitrogen, Cat# A20991), were used at 1:500 dilution. Epifluorescent images were analyzed by confocal microscopy. The WG extension phenotype was quantified by measuring the length of individual wrapping glial extension within the developing lamina at third-instar larval stage similarly as described previously [5].

### Electron microscopy

Eye-brain complexes from late third-instar larvae were dissected, fixed and sectioned similarly as described in our previous study [5]. Images were collected on a phillips techanai T12 electron microscope. The pattern of axon ensheathment was examined and quantified similarly as described in our previous study [5]. At least 3 optic stalks were quantified for each genotype.

### Cell co-aggregation

*Drosophila* S2 cells (kindly provided by Dr. David Hipfner at IRCM) were transfected with plasmid DNA (0.2 μg *actin-GAL4* and 1.8 μg *UAS* construct) for the expression of GFP, Tutl-GFP or Bdl-RFP similarly as described in our previous studies [6, 7]. Co-aggregation experiments were performed by mixing cell populations expressing different constructs similarly as described in our previous study [6]. A cell co-aggregate is defined as a cell aggregate (>20 cells) in which the percentage of each cell population (i.e. GFP- or RFP-positive cells) should be at least 15%.

### Statistical analysis

Two-tailed t-tests were used for analysis of two paired samples. One-way ANOVA were used for multiple comparisons. The difference is considered as significant when a *p* value is <0.05.

## Abbreviations

Bdl: Borderless
Htl: Heartless
PG: Perineurial glia
R cell: Photoreceptor cell
Tutl: Turtle
WG: Wrapping glia

## References

[1] Sherman DL, Brophy PJ (2005). Mechanisms of axon ensheathment and myelin growth. Nat Rev Neurosci.

[2] Franzdottir SR, Engelen D, Yuva-Aydemir Y, Schmidt I, Aho A, Klambt C (2009). Switch in FGF signalling initiates glial differentiation in the Drosophila eye. Nature.

[3] Hummel T, Attix S, Gunning D, Zipursky SL (2002). Temporal control of glial cell migration in the Drosophila eye requires gilgamesh, hedgehog, and eye specification genes. Neuron.

[4] Xie X, Gilbert M, Petley-Ragan L, Auld VJ (2014). Loss of focal adhesions in glia disrupts both glial and photoreceptor axon migration in the Drosophila visual system. Development.

[5] Cameron S, Chen Y, Rao Y (2016). Borderless regulates glial extension and axon ensheathment. Dev Biol.

[6] Cameron S, Chang WT, Chen Y, Zhou Y, Taran S, Rao Y (2013). Visual circuit assembly requires fine tuning of the novel Ig transmembrane protein Borderless. J Neurosci.

[7] Ferguson K, Long H, Cameron S, Chang WT, Rao Y (2009). The conserved Ig superfamily member Turtle mediates axonal tiling in Drosophila. J Neurosci.

[8] Bodily KD, Morrison CM, Renden RB, Broadie K (2001). A novel member of the Ig superfamily, turtle, is a CNS-specific protein required for coordinated motor control. J Neurosci.

[9] Shi SH, Cox DN, Wang D, Jan LY, Jan YN (2004). Control of dendrite arborization by an Ig family member, dendrite arborization and synapse maturation 1 (Dasm1). Proc Natl Acad Sci U S A.

[10] Doudney K, Murdoch JN, Braybrook C, Paternotte C, Bentley L, Copp AJ, Stanier P (2002). Cloning and characterization of Igsf9 in mouse and human: a new member of the immunoglobulin superfamily expressed in the developing nervous system. Genomics.

[11] Michelson AM, Gisselbrecht S, Zhou Y, Baek KH, Buff EM (1998). Dual functions of the heartless fibroblast growth factor receptor in development of the Drosophila embryonic mesoderm. Dev Genet.

[12] Al-Anzi B, Wyman RJ (2009). The Drosophila immunoglobulin gene turtle encodes guidance molecules involved in axon pathfinding. Neural Dev.

[13] Zhou Y, Cameron S, Chang WT, Rao Y (2012). Control of directional change after mechanical stimulation in Drosophila. Mol Brain.

[14] Long H, Ou Y, Rao Y, van Meyel DJ (2009). Dendrite branching and self-avoidance are controlled by Turtle, a conserved IgSF protein in Drosophila. Development.

[15] Woo J, Kwon SK, Nam J, Choi S, Takahashi H, Krueger D, Park J, Lee Y, Bae JY, Lee D, Ko J, Kim H, Kim MH, Bae YC, Chang S, Craig AM, Kim E (2013). The adhesion protein IgSF9b is coupled to neuroligin 2 via S-SCAM to promote inhibitory synapse development. J Cell Biol.

[16] Mishra A, Traut MH, Becker L, Klopstock T, Stein V, Klein R (2014). Genetic evidence for the adhesion protein IgSF9/Dasm1 to regulate inhibitory synapse development independent of its intracellular domain. J Neurosci.

[17] Newsome TP, Asling B, Dickson BJ (2000). Analysis of Drosophila photoreceptor axon guidance in eye-specific mosaics. Development.

[18] Garrity PA, Rao Y, Salecker I, McGlade J, Pawson T, Zipursky SL (1996). Drosophila photoreceptor axon guidance and targeting requires the dreadlocks SH2/SH3 adapter protein. Cell.

[19] Ruan W, Pang P, Rao Y (1999). The SH2/SH3 adaptor protein dock interacts with the Ste20-like kinase misshapen in controlling growth cone motility. Neuron.

